# Supplementary material for: Molecular hallmarks of excitatory and inhibitory neuronal resilience and resistance to Alzheimer’s disease
Source: bioRxiv. 2025 Jan 15:2025.01.13.632801. Preprint. [Version 1] doi: 10.1101/2025.01.13.632801 (PMC11761133; doi:10.1101/2025.01.13.632801)
Supplement: 1 [file NIHPP2025.01.13.632801V1-supplement-1.pdf]

Supplementary figures

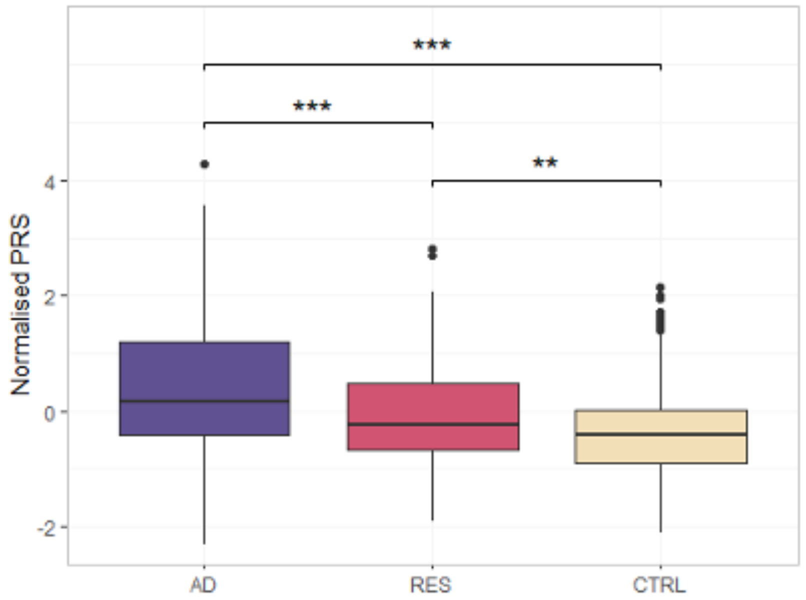

Figure S1. Sample classification details and cognitive resilience relative to AD polygenic risk.

(A) AD polygenic risk scores (AD-PRS) were calculated for each subject from ROSMAP with genetic data available. The central horizontal line of the box plots depicts the median, and the

bioRxiv preprint doi: https://doi.org/10.1101/2025.01.13.632801; this version posted January 15, 2025. The copyright holder for this preprint (which was not certified by peer review) is the author/funder, who has granted bioRxiv a license to display the preprint in perpetuity. It is made available under aCC-BY 4.0 International license.

lower and upper hinges correspond to the first and third quartiles (the 25th and 75th percentiles). The circles represent outliers. Bonferroni adjusted P values.

** Adj-P < 0.01, *** Adj-P < 0.001.

CTRL: Control, AD: Alzheimer's disease, RES: Resilient.

bioRxiv preprint doi: https://doi.org/10.1101/2025.01.13.632801; this version posted January 15, 2025. The copyright holder for this preprint (which was not certified by peer review) is the author/funder, who has granted bioRxiv a license to display the preprint in perpetuity. It is made available under aCC-BY 4.0 International license.

1502

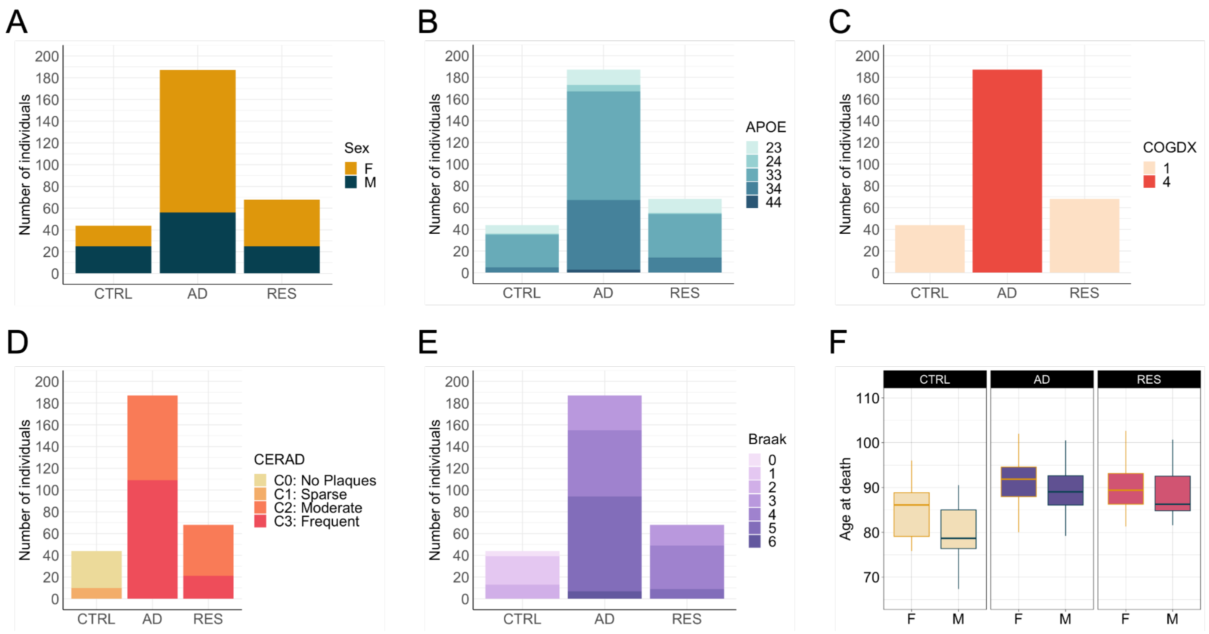

Figure S2. Characteristics of bulk RNAseq ROSMAP subjects used in this study (DLPFC).

(A) Sex distribution.

(B) *APOE* genotype distribution.

(C) Clinical consensus diagnosis of cognitive status at the time of death (final consensus cognitive diagnosis, “cogdx” variable from RUSH Alzheimer's Disease Center (RADC) Research Resource Sharing Hub).

(D) Consortium to Establish a Registry for Alzheimer's Disease (CERAD) score.

(E) Braak stage distribution.

(F) Age distribution, by sex. The central horizontal line of the box plots depicts the median, and the lower and upper hinges correspond to the first and third quartiles (the 25th and 75th percentiles).

CTRL: Control, AD: Alzheimer’s disease, RES: Resilient. F: females, M: males.

1503

bioRxiv preprint doi: https://doi.org/10.1101/2025.01.13.632801; this version posted January 15, 2025. The copyright holder for this preprint (which was not certified by peer review) is the author/funder, who has granted bioRxiv a license to display the preprint in perpetuity. It is made available under aCC-BY 4.0 International license.

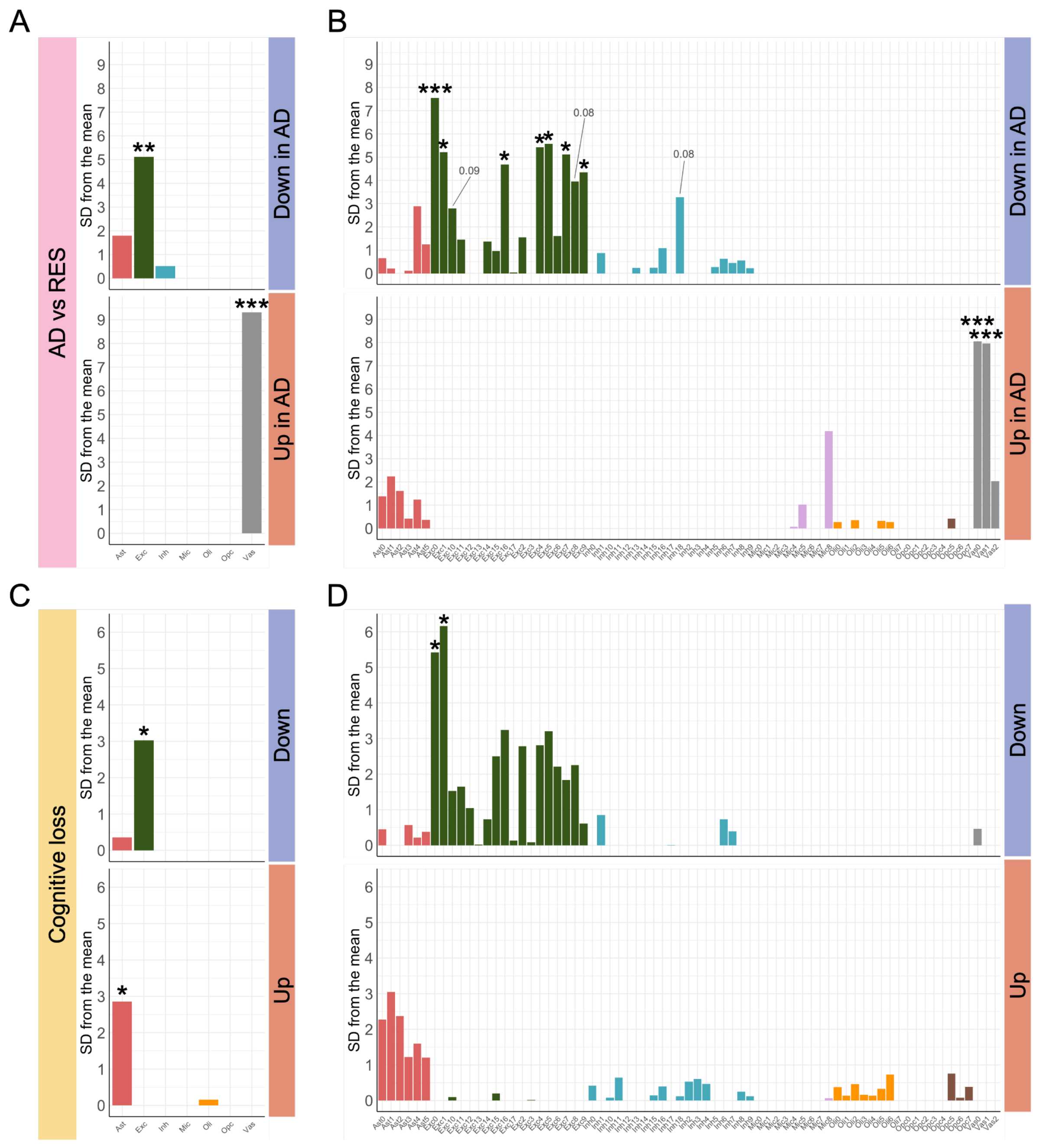

Figure S3. Expression weighted cellular enrichment results for differentially expressed genes in bulk RNAseq data from DLPFC.

(A-B) Cellular enrichment of genes uniquely differentially expressed in ADvsRES (61 DEGs) in major cell types (A) and cell subtypes (B) identified in the DLPFC.

bioRxiv preprint doi: https://doi.org/10.1101/2025.01.13.632801; this version posted January 15, 2025. The copyright holder for this preprint (which was not certified by peer review) is the author/funder, who has granted bioRxiv a license to display the preprint in perpetuity. It is made available under aCC-BY 4.0 International license.

(B-C) Cellular enrichment for genes associated with cognitive decline in major cell types (A) and cell subtypes (B). Down (top): genes down-regulated with cognitive decline; Up (bottom): genes up-regulated with cognitive decline.

Y-axis shows the number of standard deviations from the bootstrap mean, associated with the enrichment of the stated cell type in the gene list investigated. Stars represent adjusted P < 0.05 (Bonferroni correction).

bioRxiv preprint doi: https://doi.org/10.1101/2025.01.13.632801; this version posted January 15, 2025. The copyright holder for this preprint (which was not certified by peer review) is the author/funder, who has granted bioRxiv a license to display the preprint in perpetuity. It is made available under aCC-BY 4.0 International license.

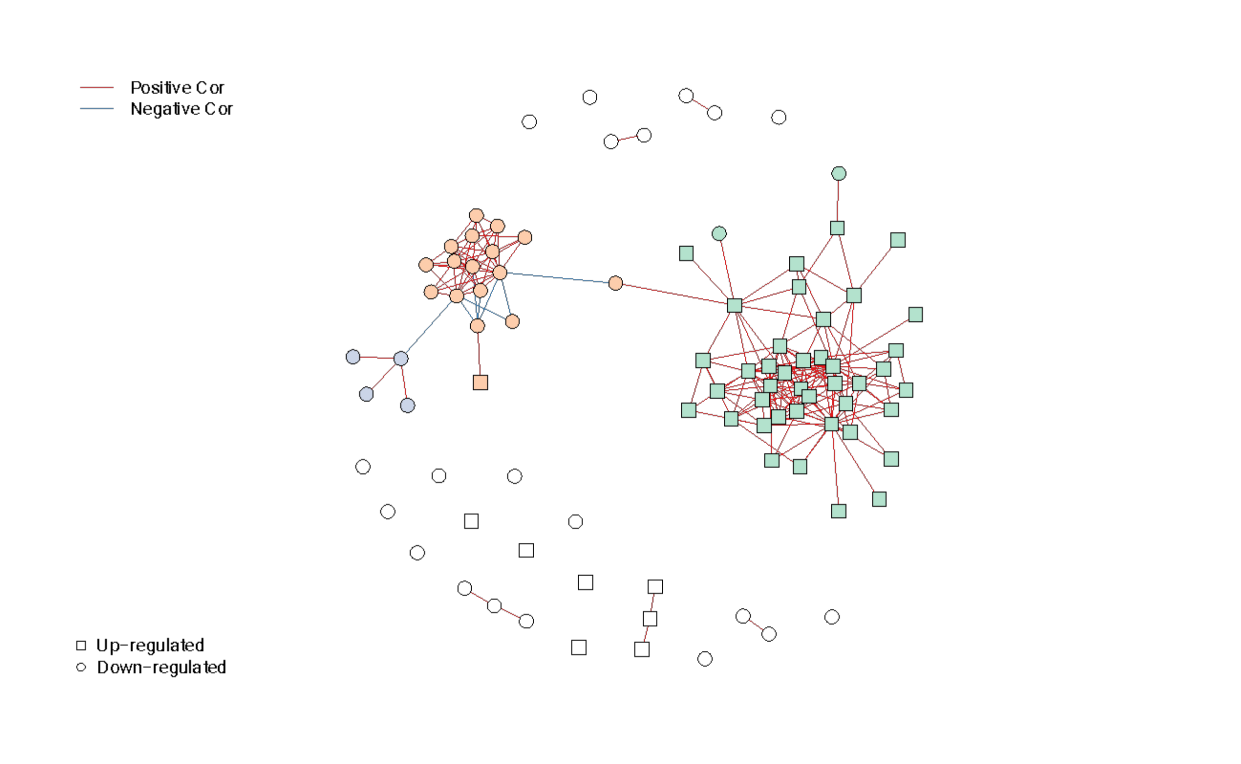

Figure S4. Resulting clusters from pathway activity analysis for ADvsRES. Nodes represent pathways with significant dysregulation of activity (q-value < 0.1) in ADvsRES. Out of a total of 99 dysregulated pathways between ADvsRES, 62 pathways organized in two unsupervised clusters of expression. Node shapes denote up-regulation and down-regulation in ADvsRES. Edges represent canonical co-expression of pathways based on the Pathway Co-expression Network background. Pathway activity profiles were determined using the PanomiR software package. Pathway dysregulation p-values were determined using the Limma package's linear regression models contrasting between ADvsRES and accounting for confounding covariates such as age, batch, and RNA integrity number.

bioRxiv preprint doi: https://doi.org/10.1101/2025.01.13.632801; this version posted January 15, 2025. The copyright holder for this preprint (which was not certified by peer review) is the author/funder, who has granted bioRxiv a license to display the preprint in perpetuity. It is made available under aCC-BY 4.0 International license.

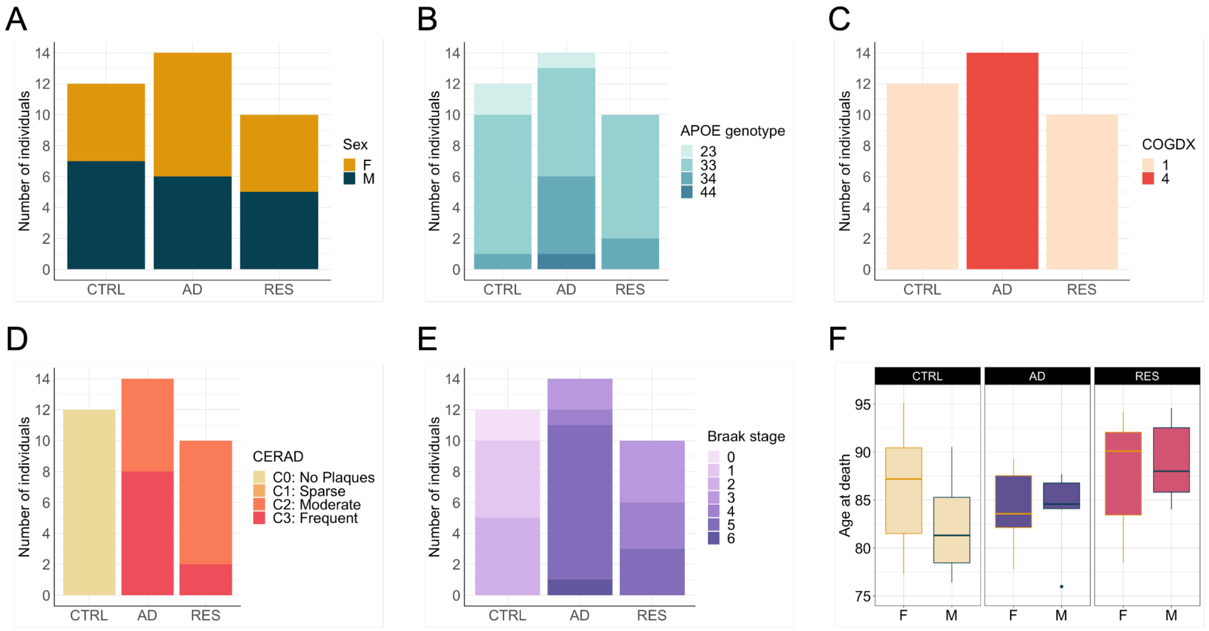

Figure S5. Characteristics of ROSMAP subjects with snRNAseq from the dorsolateral prefrontal cortex (DLPFC) used in this study.

(A) Sex distribution.

(B) APOE genotype distribution.

(C) Clinical consensus diagnosis of cognitive status at time of death (final consensus cognitive diagnosis, “cogdx” variable from RUSH Alzheimer's Disease Center (RADC) Research Resource Sharing Hub).

(D) Consortium to Establish a Registry for Alzheimer's Disease (CERAD) score.

(E) Braak stage distribution.

(F) Age distribution, by sex. The central horizontal line of the box plots depicts the median, and the lower and upper hinges correspond to the first and third quartiles (the 25th and 75th percentiles). Circles represent outliers.

CTRL: Control, AD: Alzheimer’s disease, RES: Resilient. F: females, M: males.

bioRxiv preprint doi: https://doi.org/10.1101/2025.01.13.632801; this version posted January 15, 2025. The copyright holder for this preprint (which was not certified by peer review) is the author/funder, who has granted bioRxiv a license to display the preprint in perpetuity. It is made available under aCC-BY 4.0 International license.

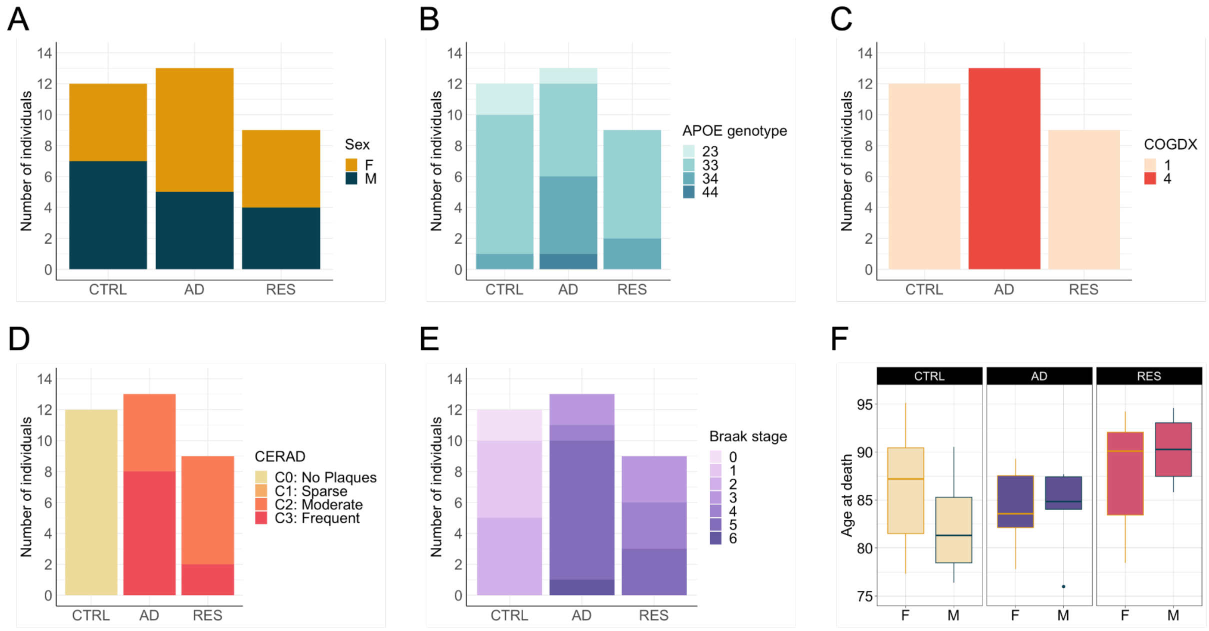

Figure S6. Characteristics of ROSMAP subjects with snRNAseq from the entorhinal cortex (EC) used in this study.

(A) Sex distribution.

(B) APOE genotype distribution.

(C) Clinical consensus diagnosis of cognitive status at time of death (final consensus cognitive diagnosis, “cogdx” variable from RUSH Alzheimer's Disease Center (RADC) Research Resource Sharing Hub).

(D) Consortium to Establish a Registry for Alzheimer's Disease (CERAD) score.

(E) Braak stage distribution.

(F) Age distribution, by sex. The central horizontal line of the box plots depicts the median, and the lower and upper hinges correspond to the first and third quartiles (the 25th and 75th percentiles). Circles represent outliers.

CTRL: Control, AD: Alzheimer’s disease, RES: Resilient. F: females, M: males.

bioRxiv preprint doi: https://doi.org/10.1101/2025.01.13.632801; this version posted January 15, 2025. The copyright holder for this preprint (which was not certified by peer review) is the author/funder, who has granted bioRxiv a license to display the preprint in perpetuity. It is made available under aCC-BY 4.0 International license.

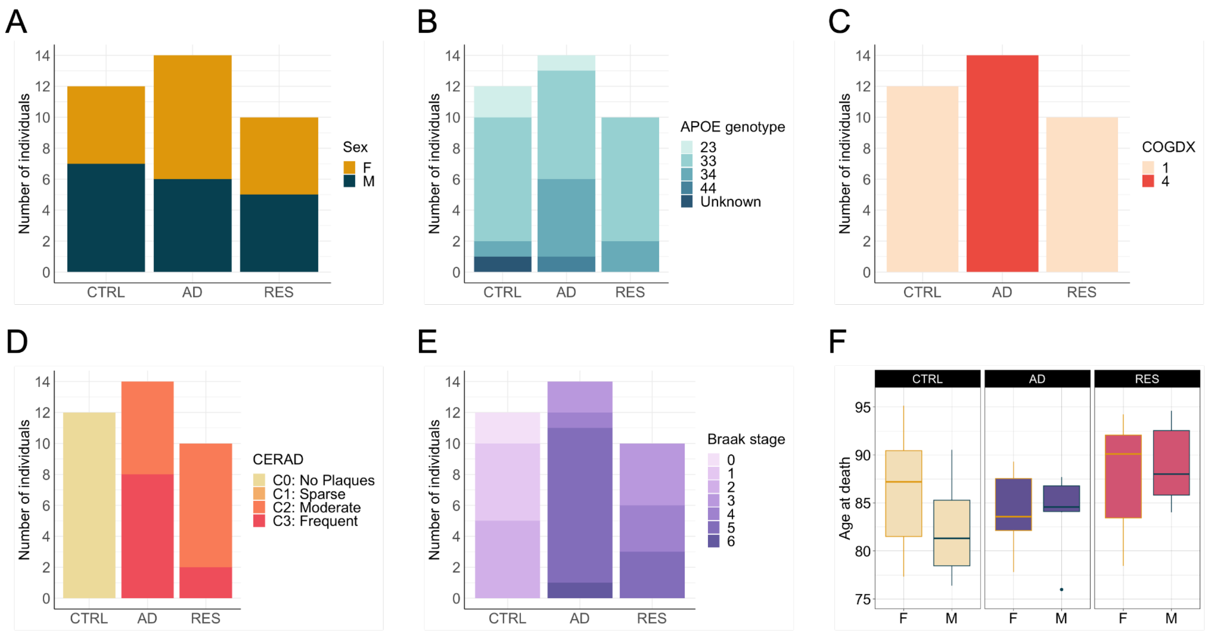

Figure S7. Characteristics of ROSMAP subjects with snRNAseq from the hippocampus (HC) used in this study.

(A) Sex distribution.

(B) APOE genotype distribution.

(C) Clinical consensus diagnosis of cognitive status at time of death (final consensus cognitive diagnosis, “cogdx” variable from RUSH Alzheimer's Disease Center (RADC) Research Resource Sharing Hub).

(D) Consortium to Establish a Registry for Alzheimer's Disease (CERAD) score.

(E) Braak stage distribution.

(F) Age distribution, by sex. The central horizontal line of the box plots depicts the median, and the lower and upper hinges correspond to the first and third quartiles (the 25th and 75th percentiles). Circles represent outliers.

CTRL: Control, AD: Alzheimer’s disease, RES: Resilient. F: females, M: males.

bioRxiv preprint doi: https://doi.org/10.1101/2025.01.13.632801; this version posted January 15, 2025. The copyright holder for this preprint (which was not certified by peer review) is the author/funder, who has granted bioRxiv a license to display the preprint in perpetuity. It is made available under aCC-BY 4.0 International license.

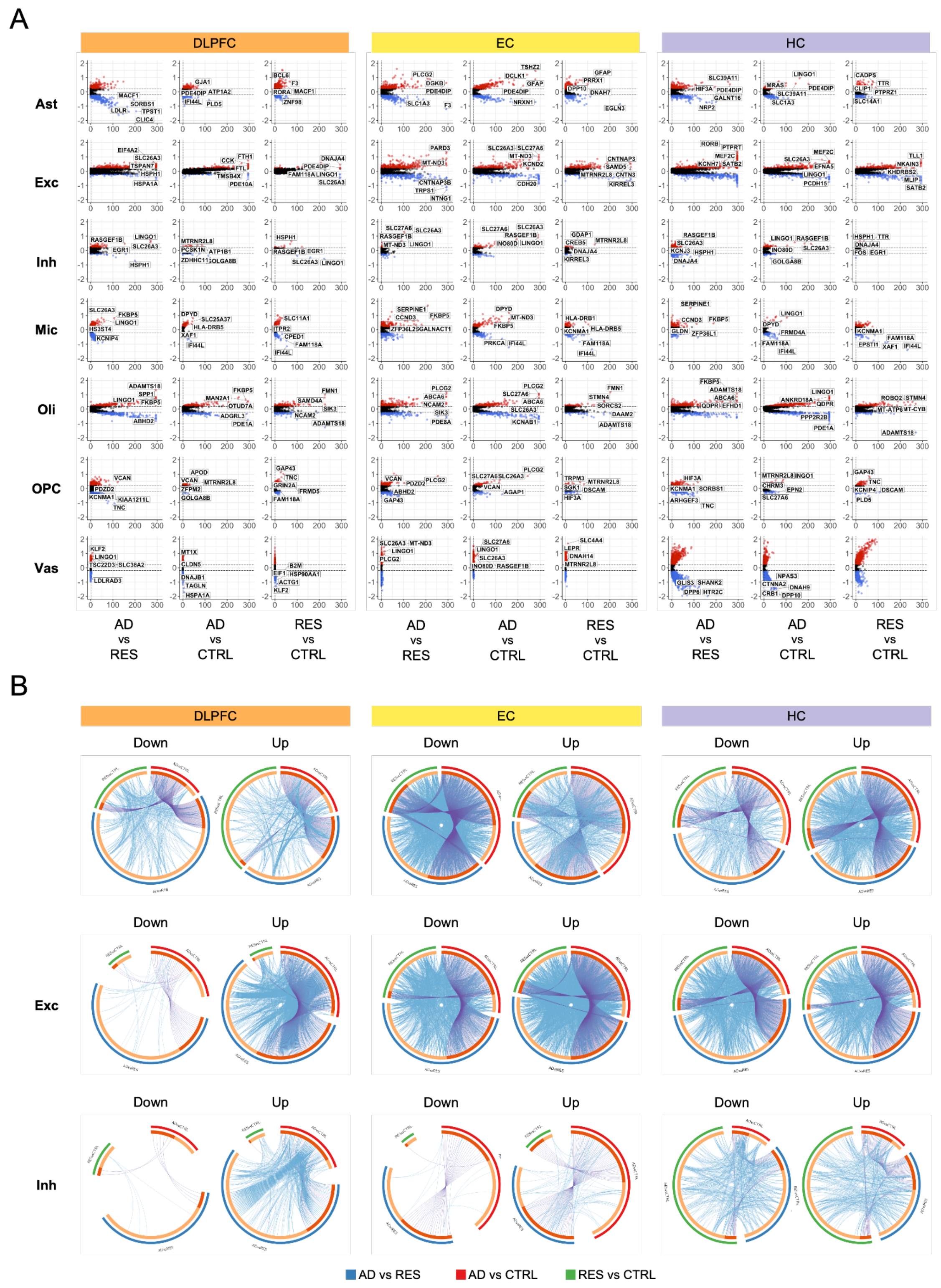

Figure S8. Cell-specific transcriptomic changes in AD resilience.

bioRxiv preprint doi: https://doi.org/10.1101/2025.01.13.632801; this version posted January 15, 2025. The copyright holder for this preprint (which was not certified by peer review) is the author/funder, who has granted bioRxiv a license to display the preprint in perpetuity. It is made available under aCC-BY 4.0 International license.

(A) Volcano plots showing significantly (Bonferroni-corrected P < 0.1) differentially expressed genes (DEGs) in each brain region tested (DLPFC, EC, and HC) in ADvsRES, ADvsCTRL, and RESvsCTRL. DEGs with log2FC < -0.2 are highlighted in blue, and DEGs with log2FC > 0.2 are highlighted in red. The horizontal lines represent Bonferroni-adjusted P = 0.1.

(B) Circular plots showing overlapping genes and ontologies from each comparison for excitatory and inhibitory neurons from each brain region investigated. Each outer arc represents the identity of each gene list (blue: ADvsRES, red: ADvsCTRL, green: RESvsCTRL). Each inner arc shows the genes that are shared by multiple lists in dark orange and genes that are unique to that gene list in light orange. Purple lines link the same gene, shared by multiple lists. Blue lines represent genes that fall under the same ontology term. Gene ontology enrichment analysis was performed using Metascape.

CTRL: Control, AD: Alzheimer's disease, RES: Resilient.

Ast: Astrocytes, Exc: Excitatory neurons, Inh: Inhibitory neurons, Mic & Imm: Microglia and immune cells, Oli: Oligodendrocytes, OPC: Oligodendrocyte progenitor cells, Vas & Epi: Vascular and epithelial cells.

DLPFC: Dorsolateral prefrontal cortex, EC: Entorhinal cortex, HC: Hippocampus.

bioRxiv preprint doi: https://doi.org/10.1101/2025.01.13.632801; this version posted January 15, 2025. The copyright holder for this preprint (which was not certified by peer review) is the author/funder, who has granted bioRxiv a license to display the preprint in perpetuity. It is made available under aCC-BY 4.0 International license.

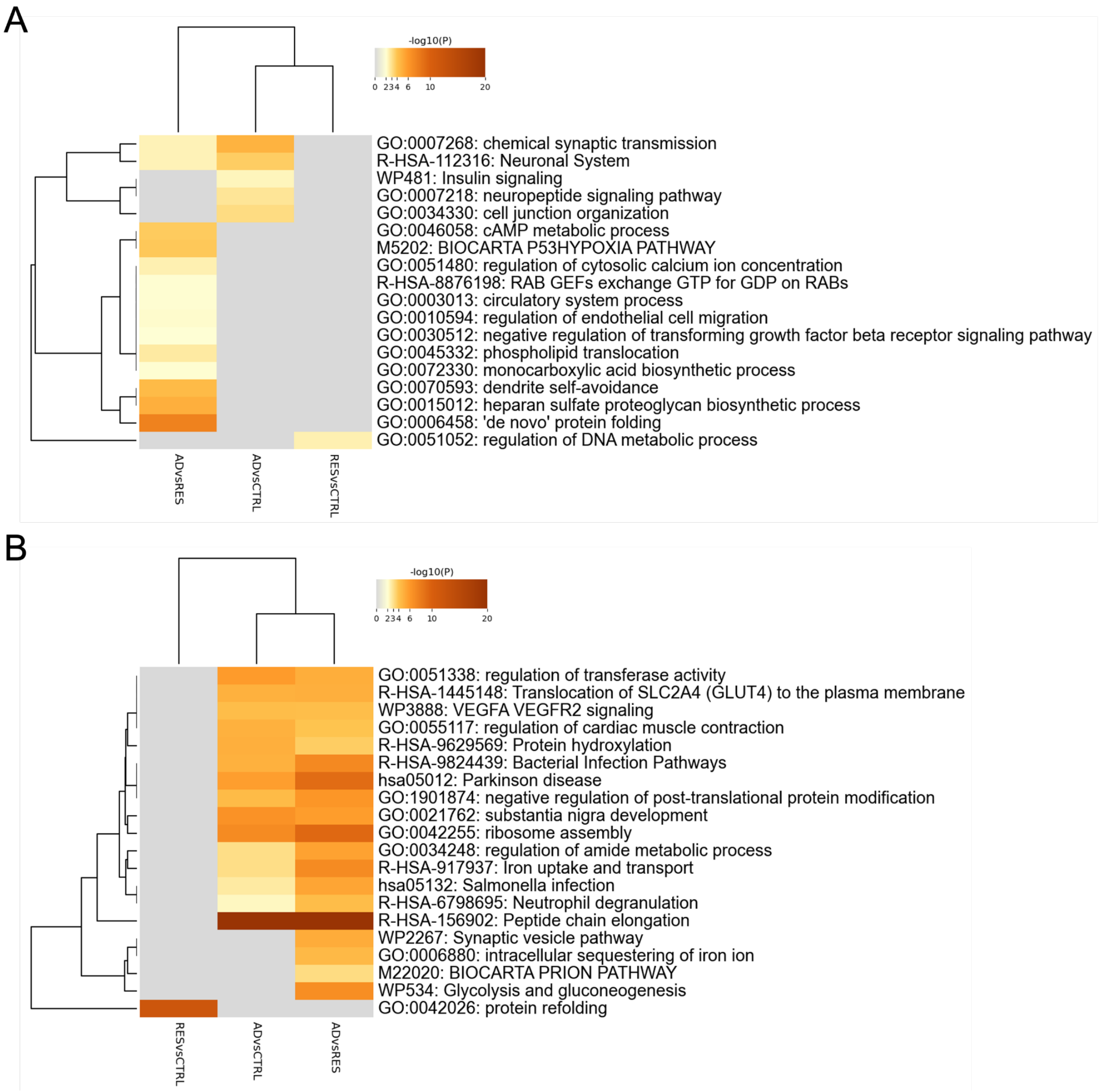

Figure S9. Significant terms from gene ontology enrichment analysis for DEGs identified in excitatory neurons from the DLPFC.

(A-B) Heatmaps showing enriched gene ontology clusters across gene lists from each of the three comparisons (ADvsRES, ADvsCTRL, RESvsCTRL). The cells in each heatmap are colored by their respective p-values, with gray cells indicating a lack of enrichment for that term in the corresponding gene list. The terms with the best p-values within each cluster are displayed in the dendrogram. Cumulative hypergeometric p-values and enrichment factors were calculated and used for filtering. Significant terms were hierarchically clustered into a

bioRxiv preprint doi: https://doi.org/10.1101/2025.01.13.632801; this version posted January 15, 2025. The copyright holder for this preprint (which was not certified by peer review) is the author/funder, who has granted bioRxiv a license to display the preprint in perpetuity. It is made available under aCC-BY 4.0 International license.

tree based on Kappa-statistical similarities among their gene memberships, with 0.3 kappa score applied as the threshold to cast the tree into clusters.

(A) Genes down-regulated in the first group compared to the second group for each comparison.

(B) Genes up-regulated in the first group compared to the second group for each comparison.

bioRxiv preprint doi: https://doi.org/10.1101/2025.01.13.632801; this version posted January 15, 2025. The copyright holder for this preprint (which was not certified by peer review) is the author/funder, who has granted bioRxiv a license to display the preprint in perpetuity. It is made available under aCC-BY 4.0 International license.

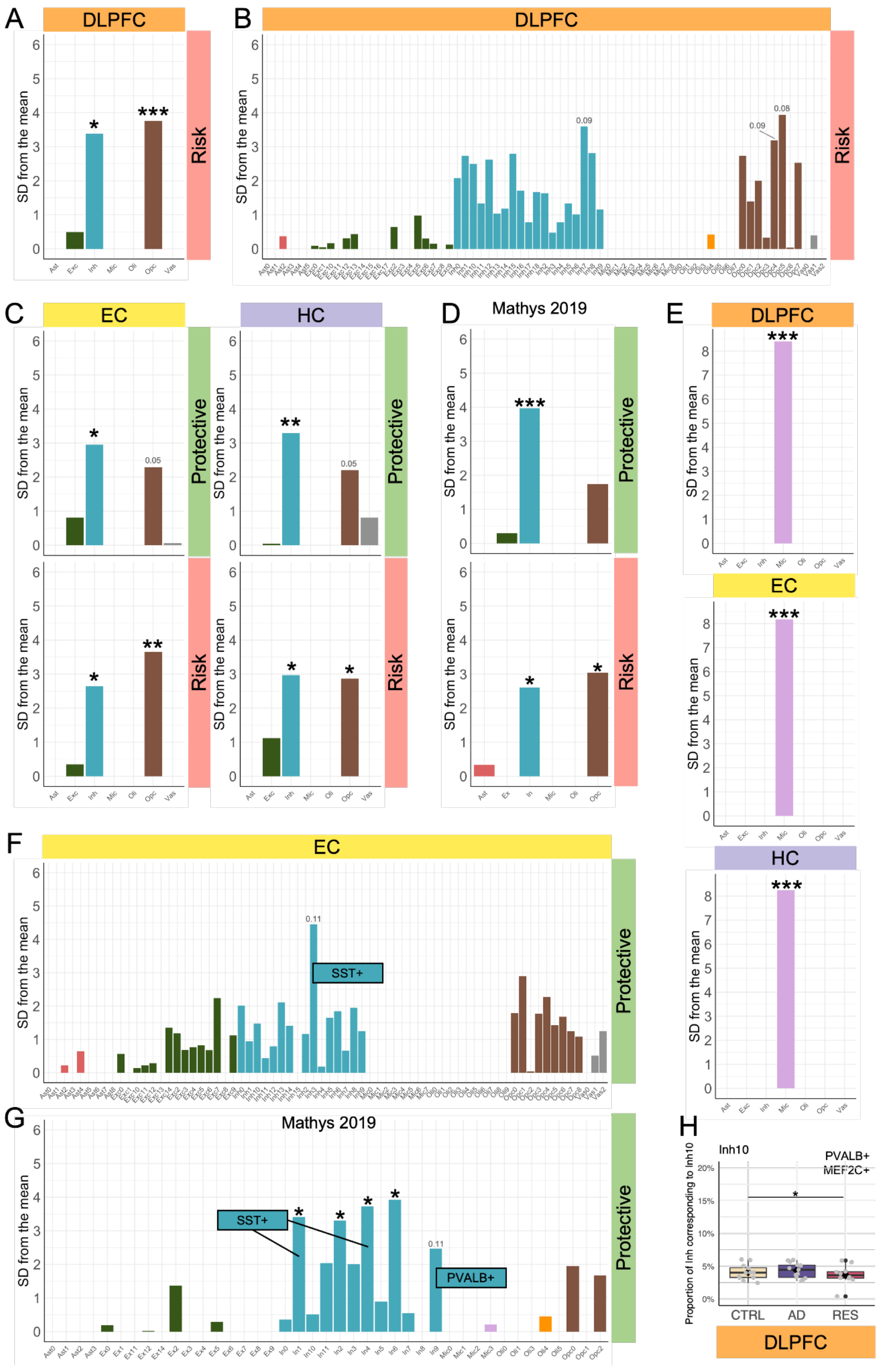

Figure S10. Expression-weighted cellular enrichment results for genes identified from rare variants.

bioRxiv preprint doi: https://doi.org/10.1101/2025.01.13.632801; this version posted January 15, 2025. The copyright holder for this preprint (which was not certified by peer review) is the author/funder, who has granted bioRxiv a license to display the preprint in perpetuity. It is made available under aCC-BY 4.0 International license.

(A) Cellular enrichment of genes identified from risk genetic rare variants in the DLPFC for major cell types.

(B) Cellular enrichment of genes identified from risk genetic rare variants in the DLPFC for cell subtypes.

(C) Cellular enrichment of genes identified from protective and risk genetic rare variants in the EC and HC for major cell types.

(D) Cellular enrichment of genes identified from protective and risk genetic rare variants in the Mathys et al. 2019 dataset (DLPC) for major cell types.

(E) Genes identified from common variants from Bellenguez et al. 2022 in major cell types.

(F) Cellular enrichment of genes identified from protective rare variants in the EC for cell subtypes.

(G) Cellular enrichment of genes identified from protective rare variants in the Mathys et al. 2019 dataset (DLPC) for cell subtypes.

(E) Cell proportion changes for PVALB+ DLPFC:Inh10.

* Adj-P < 0.05, ** Adj-P < 0.01, *** Adj-P < 0.001

CTRL: Control, AD: Alzheimer's disease, RES: Resilient.

DLPFC: Dorsolateral prefrontal cortex, EC: Entorhinal cortex, HC: Hippocampus.

bioRxiv preprint doi: https://doi.org/10.1101/2025.01.13.632801; this version posted January 15, 2025. The copyright holder for this preprint (which was not certified by peer review) is the author/funder, who has granted bioRxiv a license to display the preprint in perpetuity. It is made available under aCC-BY 4.0 International license.

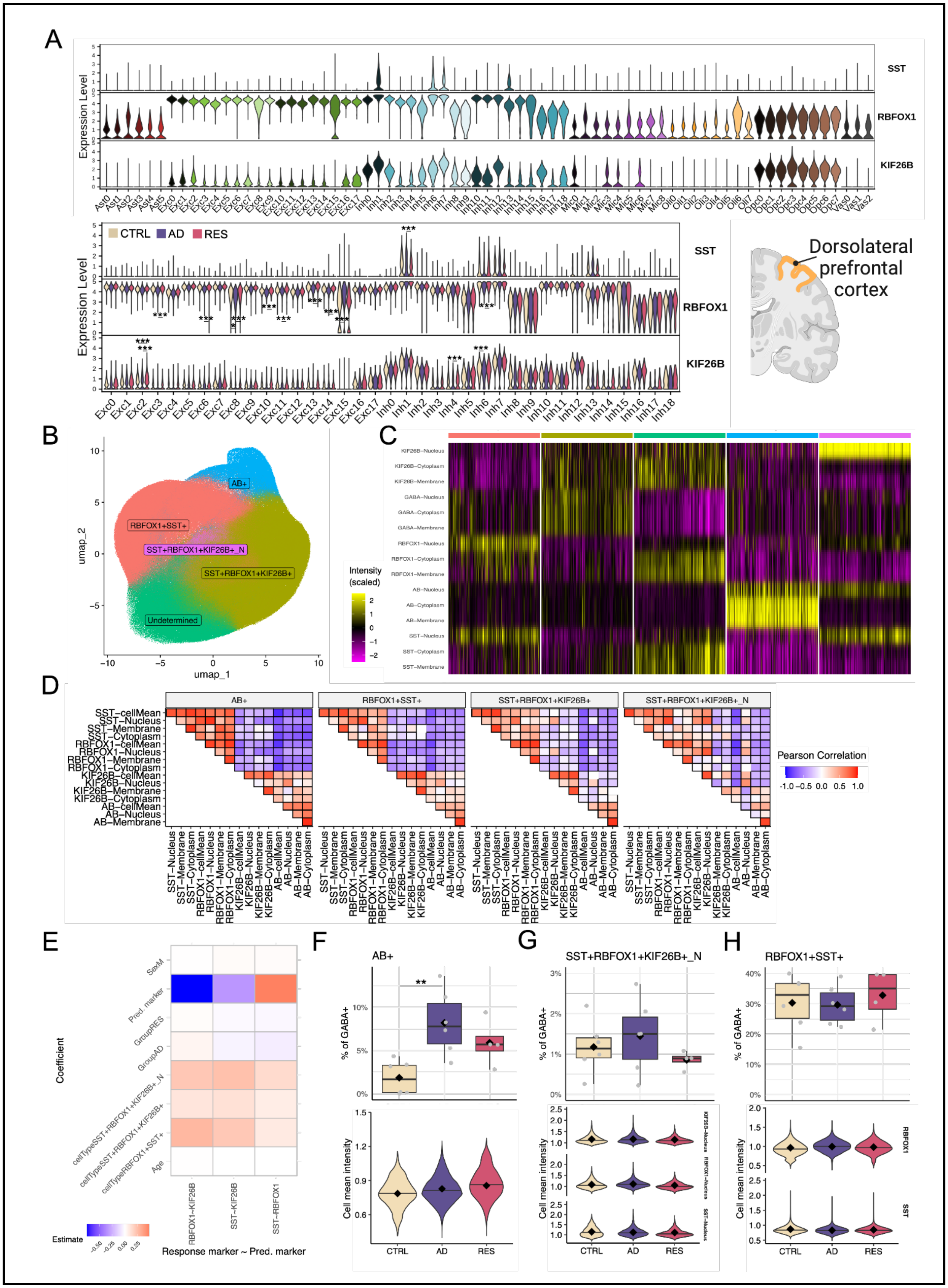

bioRxiv preprint doi: https://doi.org/10.1101/2025.01.13.632801; this version posted January 15, 2025. The copyright holder for this preprint (which was not certified by peer review) is the author/funder, who has granted bioRxiv a license to display the preprint in perpetuity. It is made available under aCC-BY 4.0 International license.

Figure S11. Gene expression distributions and protein expression assessed by mIF for SST, RBFOX1, and KIF26B.

(A) Violin plots showing gene expression levels of SST, RBFOX1, and KIF26B for each subtype (top) and neuronal subtypes by diagnostic group (bottom) in the DLPFC (related to **Figure 3L**).

(B) UMAP showing the populations identified in the DLPFC in the BIDMC cohort from multiplex immunofluorescence (mIF) staining for GABA, SST, RBFOX1, KIF26B, and Aβ. $N_{subjects}$ = 16 (6 CTRL, 6 AD, 4 RES), N_{cells} = 1310803 (CTRL = 465007, AD = 559794, RES = 286002 cells).

(C) Heatmap of normalized intensity of protein markers in a random subset of 500 cells from each cell type (indicated by the top color bar). Cell type labels shown in (B).

(D) Correlation of inhibitory neuronal targets normalized intensity across identified cell subpopulations. Each square is colored by the Pearson correlation coefficient corresponding to the pair of markers indicated in the x and y axes. Solid black squares indicate significant correlations at an FDR of 0.05.

(E) Coefficients of predictors used in linear models of marker intensities.

(F-H) Box plots (top) showing the results for differential cell proportions of clusters shown in **(B)** and violin plots (bottom) showing the distribution of their corresponding cell-level protein intensities (normalized immunofluorescence levels) in each population. Diamonds show the grand mean of subject-level mean normalized intensities across cells.

CTRL: Control, AD: Alzheimer's disease, RES: Resilient.

DLPFC: Dorsolateral prefrontal cortex.

* Adj-P < 0.05, ** Adj-P < 0.01, *** Adj-P < 0.001.

bioRxiv preprint doi: https://doi.org/10.1101/2025.01.13.632801; this version posted January 15, 2025. The copyright holder for this preprint (which was not certified by peer review) is the author/funder, who has granted bioRxiv a license to display the preprint in perpetuity. It is made available under aCC-BY 4.0 International license.

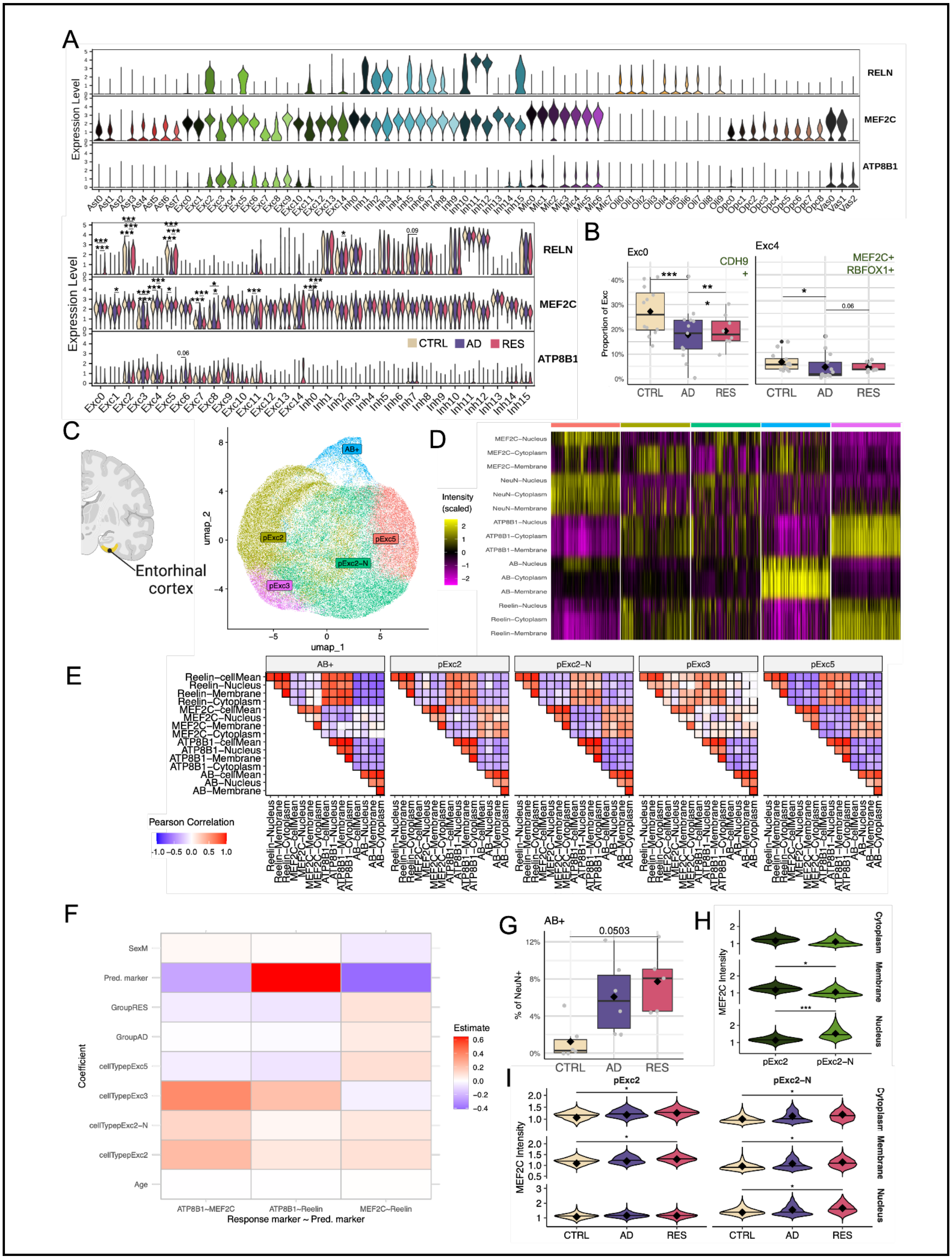

bioRxiv preprint doi: https://doi.org/10.1101/2025.01.13.632801; this version posted January 15, 2025. The copyright holder for this preprint (which was not certified by peer review) is the author/funder, who has granted bioRxiv a license to display the preprint in perpetuity. It is made available under aCC-BY 4.0 International license.

Figure S12. Significant cell proportion changes in vulnerable excitatory neurons and gene expression dynamics in excitatory neurons associated with resilience.

(A) Violin plots showing gene expression levels (snRNAseq) for RELN, MEF2C, and ATP8B1 for all subtypes (top) and neuronal subtypes by group (bottom) in the EC (related to **Figure 4**). Cell proportion changes in additional vulnerable excitatory subtypes from the entorhinal cortex, as reported by Leng et al., 2021.

(B) Cell proportion changes for CDH9+ EC:Exc0 cells (left) and cell proportion changes for RBFOX1+ $MEF2C_{high}$ EC:Exc4 (right)

(**C**) UMAP showing the populations identified in the EC in the BIDMC cohort from multiplex immunofluorescence (mIF) staining for NeuN, MEF2C, ATP8B1, RELN, and Aβ. $N_{subjects}$ = 16 (6 CTRL, 6 AD, 5 RES), N_{cells} = 81549 cells.

(D) Heatmap of normalized intensity of protein markers in a random subset of 500 cells from each cell type (indicated by the top color bar). Cell type labels shown in **(B).**

(E) Correlation of inhibitory neuronal targets normalized intensity across identified cell subpopulations. Each square is colored by the Pearson correlation coefficient corresponding to the pair of markers indicated in the x and y axes. Solid black squares indicate significant correlations at an FDR of 0.05.

(F) Coefficients of predictors used in linear models of marker intensities.

(G) Box plots showing cell proportion changes in the cluster AB+ shown in **(B)**, corresponding to neurons expressing high protein levels of Aβ.

(H) Violin plots showing protein expression (normalized immunofluorescence intensity levels) of MEF2C in different compartments (cytoplasm, membrane, and nucleus) for the clusters pExc2 and pExc2-N ($MEF2C_{high}$ ATP8B1+ RELN+) shown in **(D)**.

(I) Violin plots showing distributions of protein expression (normalized immunofluorescence intensity levels) of MEF2C in different compartments (cytoplasm, membrane, and nucleus) for

bioRxiv preprint doi: https://doi.org/10.1101/2025.01.13.632801; this version posted January 15, 2025. The copyright holder for this preprint (which was not certified by peer review) is the author/funder, who has granted bioRxiv a license to display the preprint in perpetuity. It is made available under aCC-BY 4.0 International license.

the clusters pExc2 and pExc2-N ($MEF2C_{high}$ ATP8B1+ RELN+) by diagnostic group (CTRL, AD, RES). In **(H)** and **(I)** stars indicate significance level based on nominal P values of a Wilcoxon test performed on the subject-level means. Diamonds show the grand mean of subject-level mean normalized intensities across cells.

CTRL: Control, AD: Alzheimer’s disease, RES: Resilient.

EC: Entorhinal cortex.

* Adj-P < 0.05, ** Adj-P < 0.01, *** Adj-P < 0.001

bioRxiv preprint doi: https://doi.org/10.1101/2025.01.13.632801; this version posted January 15, 2025. The copyright holder for this preprint (which was not certified by peer review) is the author/funder, who has granted bioRxiv a license to display the preprint in perpetuity. It is made available under aCC-BY 4.0 International license.

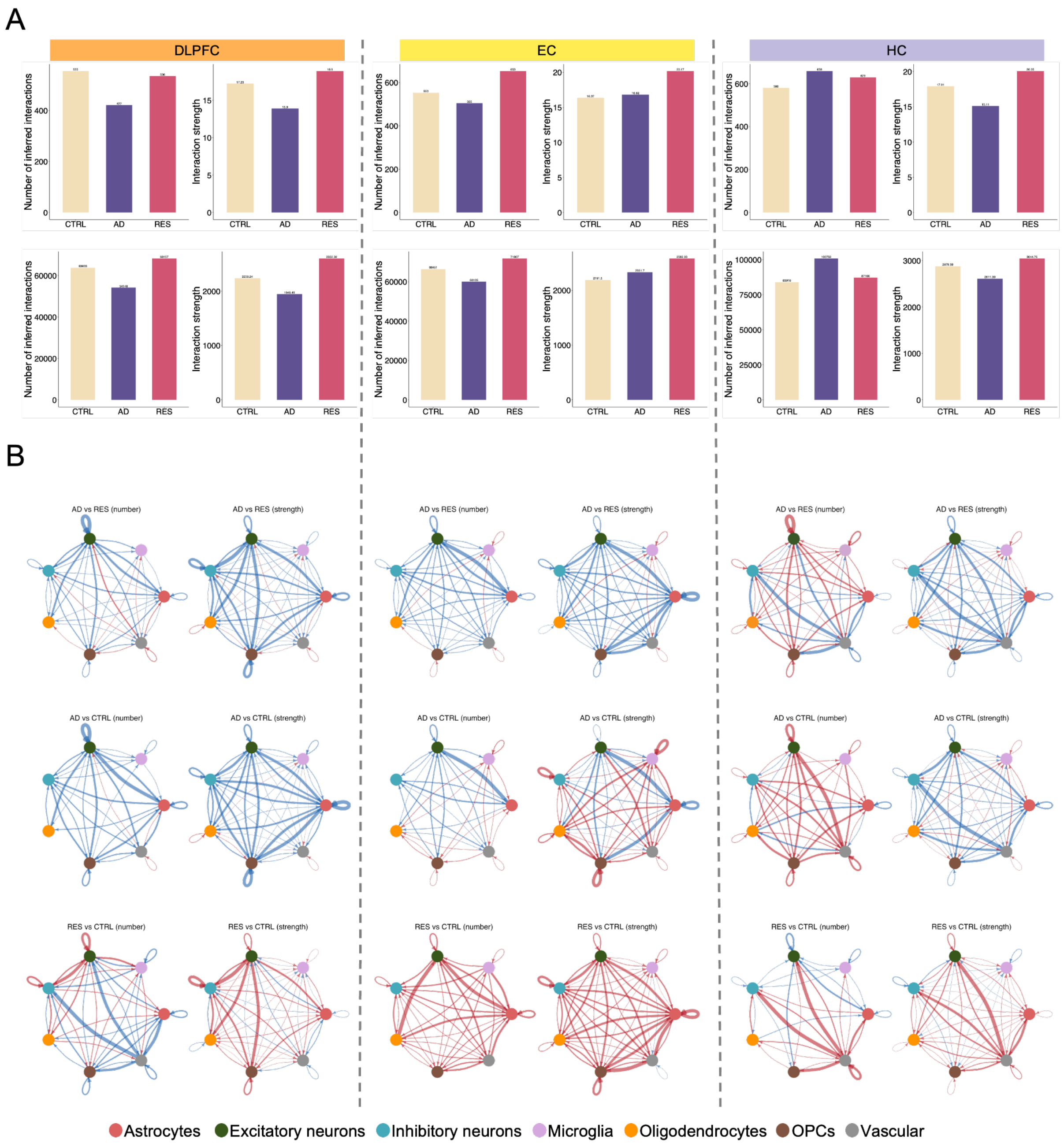

Figure S13. An increase in the number of events in cell-cell communication in resilience.

(A) Bar plots showing the inferred number and strength of ligand-receptor interactions in each group for major cell populations (top) and cell subpopulations (bottom) in each brain region.

bioRxiv preprint doi: https://doi.org/10.1101/2025.01.13.632801; this version posted January 15, 2025. The copyright holder for this preprint (which was not certified by peer review) is the author/funder, who has granted bioRxiv a license to display the preprint in perpetuity. It is made available under aCC-BY 4.0 International license.

(B) Differential number and strength of cell-cell interactions for each comparison. Arrows represent direction of interactions, from source to target major cell class. Blue represents a decrease in the number or strength of interactions between any two cells in the first compared to the second diagnostic group, and red represents an increase.

CTRL: Control, AD: Alzheimer's disease, RES: Resilient.

bioRxiv preprint doi: https://doi.org/10.1101/2025.01.13.632801; this version posted January 15, 2025. The copyright holder for this preprint (which was not certified by peer review) is the author/funder, who has granted bioRxiv a license to display the preprint in perpetuity. It is made available under aCC-BY 4.0 International license.

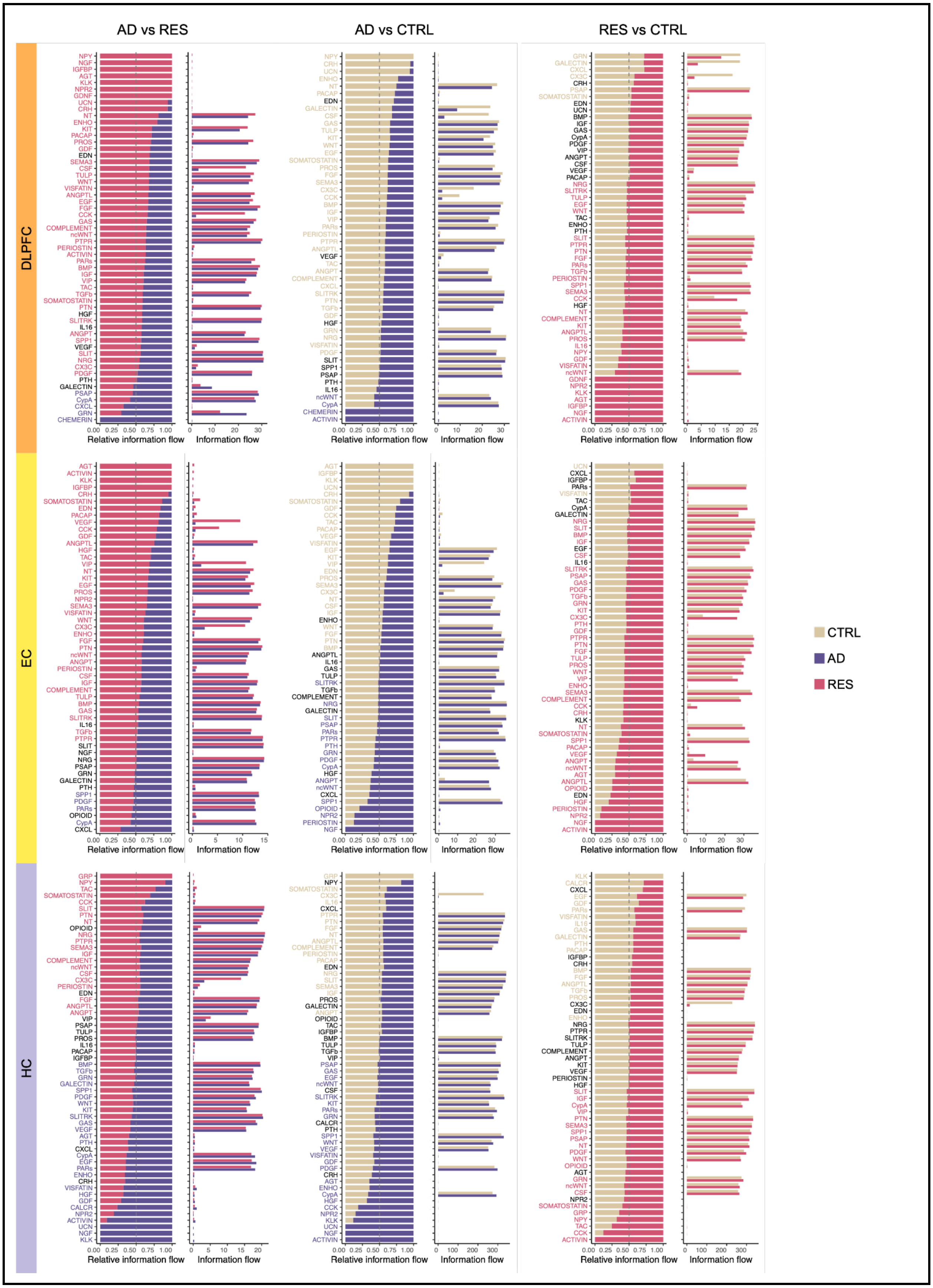

bioRxiv preprint doi: https://doi.org/10.1101/2025.01.13.632801; this version posted January 15, 2025. The copyright holder for this preprint (which was not certified by peer review) is the author/funder, who has granted bioRxiv a license to display the preprint in perpetuity. It is made available under aCC-BY 4.0 International license.

Figure S14. Significant pathways from intercellular communication analysis in cell subpopulations.

List of significant pathways in each brain region (DLPFC, EC, and HC) for each comparison (ADvsRES, ADvsCTRL, and RESvsCTRL). Colored pathway names are significantly shifted towards the group with the corresponding color. Plots on the left show the relative information flow in one diagnostic group compared to the other, and the plots on the right show the overall information flow for each signaling pathway.

CTRL: Control, AD: Alzheimer's disease, RES: Resilient.

bioRxiv preprint doi: https://doi.org/10.1101/2025.01.13.632801; this version posted January 15, 2025. The copyright holder for this preprint (which was not certified by peer review) is the author/funder, who has granted bioRxiv a license to display the preprint in perpetuity. It is made available under aCC-BY 4.0 International license.

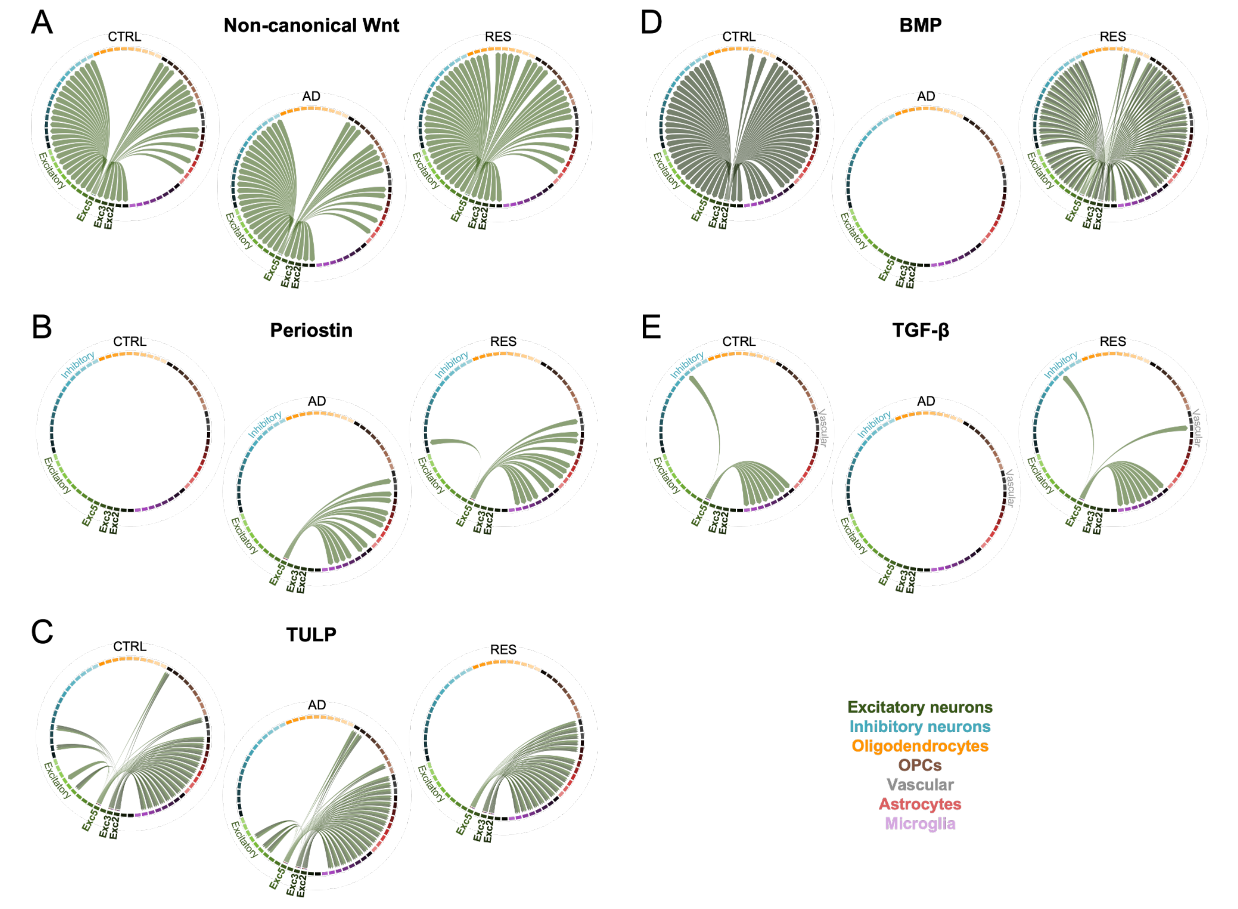

Figure S15. Signaling pathways emerging or disappearing in resilience in excitatory neuronal subpopulations from the entorhinal cortex showing a resilient phenotype.

Chord diagrams showing significant networks (Figure S15) having EC:Exc2, EC:Exc:3, or EC:Exc5 as sources.

(A) Non-canonical Wnt (source: EC:Exc5; targets: multiple subtypes of oligodendrocytes; ligand: WNT5A; receptor: MCAM).

(B) Periostin (source: EC:Exc5; target: EC:Inh1; ligand: periostin; receptor: ITGAV/ITGB5).

(C) TULP (sources: EC:Exc2, EC:Exc3, and EC:Exc5; targets: subtypes of excitatory neurons, inhibitory neurons, OPCs, and astrocytes; ligand: TUB; receptor: MERTK).

bioRxiv preprint doi: https://doi.org/10.1101/2025.01.13.632801; this version posted January 15, 2025. The copyright holder for this preprint (which was not certified by peer review) is the author/funder, who has granted bioRxiv a license to display the preprint in perpetuity. It is made available under aCC-BY 4.0 International license.

(D) BMP (source: EC:Exc5; targets: subtypes from all major cell types; ligands: GDF7 and BMP8A; receptors: BMPR1A/ACVR2A, BMPR1A/ACVR2B, BMPR1A/BMPR2, BMPR1B/ACVR2A, BMPR1B/BMPR2, ACVR1/ACVR2A, ACVR1/BMPR2, BMPR1B/ACVR2B).

(E) TGF-β (source: EC:Exc5; target: EC:Fib; ligand: TGF-β2; receptors: TGFBR1/R2 and ACVR1/TGFBR).

CTRL: Control, AD: Alzheimer's disease, RES: Resilient.

bioRxiv preprint doi: https://doi.org/10.1101/2025.01.13.632801; this version posted January 15, 2025. The copyright holder for this preprint (which was not certified by peer review) is the author/funder, who has granted bioRxiv a license to display the preprint in perpetuity. It is made available under aCC-BY 4.0 International license.

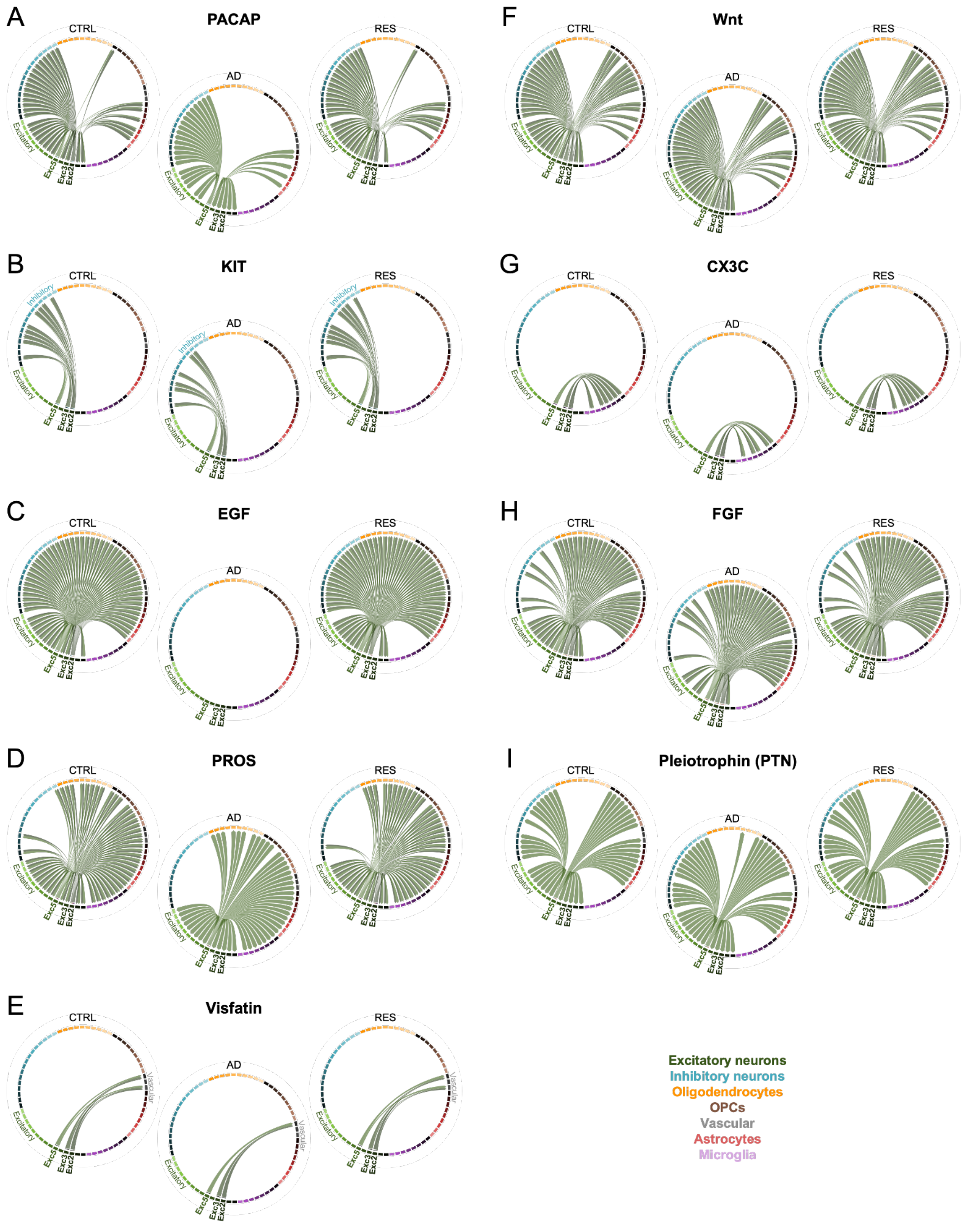

Figure S16. Signaling pathways dynamics in AD in resilience-associated excitatory neuronal subpopulations from the entorhinal cortex.

bioRxiv preprint doi: https://doi.org/10.1101/2025.01.13.632801; this version posted January 15, 2025. The copyright holder for this preprint (which was not certified by peer review) is the author/funder, who has granted bioRxiv a license to display the preprint in perpetuity. It is made available under aCC-BY 4.0 International license.

Chord diagrams showing significant networks (Figure S15) with EC:Exc2, EC:Exc:3, and EC:Exc5 as sources.

(A) PACAP (sources: EC:Exc2 and EC:Exc5; targets: subtypes of multiple major cell types; ligand: ADCYAP1; receptor: ADCYAP1R1).

(B) KIT (sources: EC:Exc2, EC:Exc:3, and EC:Exc5; target: EC:Inh6, EC:Inh10, and EC:Inh14; ligand: KITLG; receptor: KIT).

(C) EGF (source: EC:Exc2 and EC:Exc5; targets: subtypes of all major cell types except microglia; ligands: EGF and BTC; receptors: EGFR and ERBB4).

(D) PROS (source: EC:Exc2; targets: subtypes from all major cell types; ligand: PROS1; receptors: AXL, TYRO3, MERTK).

(E) Visfatin (sources: EC:Exc2, EC:Exc3, EC:Exc5; target: EC:Fib; ligand: NAMPT; receptor:ITGA5/ITGB1).

(F) Wnt (sources: EC:Exc2, EC:Exc3, EC:Exc5; targets: EC:Opc4 (disappears in AD), and EC:Exc14 (emerges in AD); ligands: WNT10B, and WNT3 (in AD only); receptor: FZD3/LRP6).

(G) CX3C (sources: EC:Exc2, EC:Exc3, EC:Exc5; target: EC:Mic2; ligand: CX3CL1; receptor: CX3CR1).

(H) FGF (sources: EC:Exc2, EC:Exc3, EC:Exc5; targets: multiple subtypes of excitatory neurons, inhibitory neurons (loss in some subtypes and gain in others), and EC:Vas0 (emerges in AD); ligands: FGF5, FGF9, and FGF17; receptors: FGFR1 and FGFR2).

(I) Pleiotrophin (source: EC:Exc5; targets: EC:Exc13 (gain), EC:Inh6 and EC:Inh8 (gain), EC:Oli6 and EC:Oli9 (gain), EC:Fib (loss), and EC:Ast2 (loss); ligand: PTN; receptors: PTPRZ1, SDC2 (loss), SDC3, NCL, ALK).

CTRL: Control, AD: Alzheimer's disease, RES: Resilient.

bioRxiv preprint doi: https://doi.org/10.1101/2025.01.13.632801; this version posted January 15, 2025. The copyright holder for this preprint (which was not certified by peer review) is the author/funder, who has granted bioRxiv a license to display the preprint in perpetuity. It is made available under aCC-BY 4.0 International license.

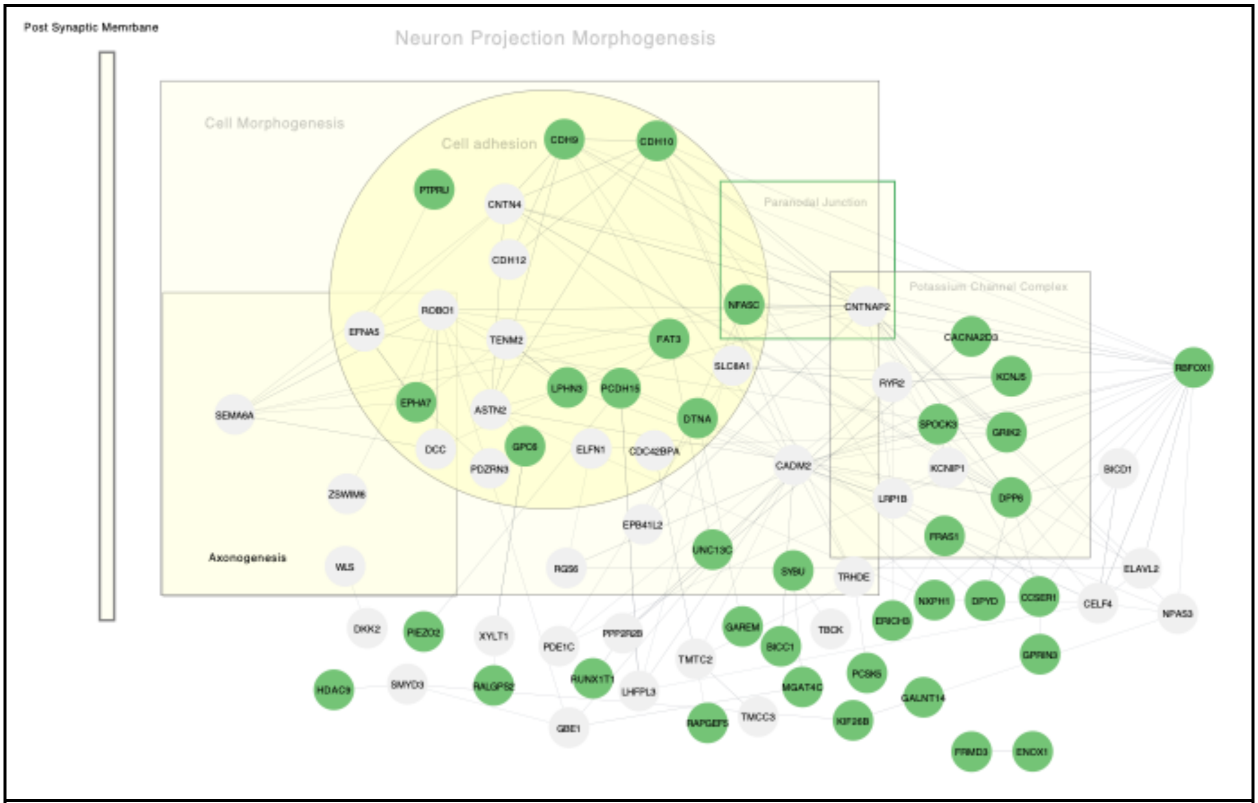

Figure S17. **StringDB network of genes associated with risk and protective rare variants also identified as marker genes for DLPFC:Inh1 neurons.**

RBFOX1 interaction partners and marker genes are enriched in cell morphogenesis, paranodal junction, potassium channel complex, and axonogenesis. All may contribute to neuron projection morphogenesis. Genes from protective variants are shown as green nodes, and genes from risk variants as gray nodes. Visualization was performed using Cytoscape 3.10.2. (NDEx access to network)

bioRxiv preprint doi: https://doi.org/10.1101/2025.01.13.632801; this version posted January 15, 2025. The copyright holder for this preprint (which was not certified by peer review) is the author/funder, who has granted bioRxiv a license to display the preprint in perpetuity. It is made available under aCC-BY 4.0 International license.

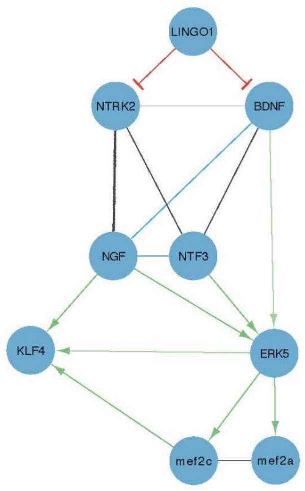

Figure S18. LINGO1 regulation of neurotrophin pathway in resilient cells. STRINGdb relationships and supporting literature were used to establish protein-protein and co-expression relationships between key members of the neurotrophin pathway. Visualization was performed using Cytoscape 3.10.2. Red - inhibition, green arrow, activation. Black, interaction, gray, imputed interaction.

Supplementary tables

Table S1. Differentially expressed genes in AD compared to cognitive resilience in bulk DLPFC tissue.

Differential gene expression analysis was performed between AD and resilient subjects (ADvsRES) using ROSMAP bulk RNAseq data. Significant differential expression was determined using adjusted p-value (FDR) < 0.1 (highlighted in red) and |FC| > 1.1 criteria. P-

bioRxiv preprint doi: https://doi.org/10.1101/2025.01.13.632801; this version posted January 15, 2025. The copyright holder for this preprint (which was not certified by peer review) is the author/funder, who has granted bioRxiv a license to display the preprint in perpetuity. It is made available under aCC-BY 4.0 International license.

values were determined using a linear regression model accounting for confounding covariates such as sequencing batch, RNA integrity number, and postmortem interval. The column "Direction" shows significant upregulation ("UP") or downregulation ("DOWN") in AD versus resilience as defined above. The column "Unique ADvsRES" reports genes that were differentially expressed in ADvsRES and not in ADvsCTRL or RESvsCTRL. The column "Cognitive loss" reports genes also associated with loss of cognition (related with **Table S5**).

Table S2. Differentially expressed genes in AD compared to cognitively healthy controls in bulk DLPFC tissue.

Differential gene expression analysis was performed between AD and control subjects (ADvsCTRL) using ROSMAP bulk RNAseq data. Significant differential expression was determined using adjusted p-value (FDR) < 0.1 (highlighted in red) and |FC| > 1.1 criteria. P-values were determined using a linear regression model accounting for confounding covariates such as sequencing batch, RNA integrity number, and postmortem interval. The column "Direction" shows significant upregulation ("UP") or downregulation ("DOWN") in AD versus controls as defined above.

Table S3. Differentially expressed genes in resilient subjects compared to cognitively healthy controls in bulk DLPFC tissue.

Differential gene expression analysis was performed between resilient and control subjects (RESvsCTRL) using ROSMAP bulk RNAseq data. Significant differential expression was determined using adjusted p-value (FDR) < 0.1 (highlighted in red) and |FC| > 1.1 criteria. P-values were determined using a linear regression model accounting for confounding covariates such as sequencing batch, RNA integrity number, and postmortem interval. The column "Direction" shows significant upregulation ("UP") or downregulation ("DOWN") in resilience versus controls as defined above.

bioRxiv preprint doi: https://doi.org/10.1101/2025.01.13.632801; this version posted January 15, 2025. The copyright holder for this preprint (which was not certified by peer review) is the author/funder, who has granted bioRxiv a license to display the preprint in perpetuity. It is made available under aCC-BY 4.0 International license.

Table S4. Differentially expressed genes in AD compared to subjects classified as presymptomatic in bulk DLPFC tissue.

Differential gene expression analysis was performed between AD and presymptomatic subjects (ADvsPRE) using ROSMAP bulk RNAseq data. Significant differential expression was determined using adjusted p-value (FDR) < 0.1 (highlighted in red) and |FC| > 1.1 criteria. P-values were determined using a linear regression model accounting for confounding covariates such as sequencing batch, RNA integrity number, and postmortem interval. The column “Direction” shows significant upregulation (“UP”) or downregulation (“DOWN”) in AD versus presymptomatic as defined above.

Table S5. Genes associated with loss of cognition from bulk RNAseq data.

The association of gene expression profiles with loss of cognition was analyzed using a proportional odds model (POM) for ordinal categorical data analysis applied to ROSMAP bulk RNAseq data. Gene expression levels were adjusted for confounding covariates prior to performing POM. The POM analysis was applied to the subjects with either no cognitive impairment, mild cognitive impairment, or AD dementia. The POM analyzed cognitive impairment as a function of gene expression and pathology status (plaque and tangle stages). P-values represent the significance of the association of each gene with cognitive status. The column "Direction" denotes whether the expression of a gene positively or negatively correlates with loss of cognition. The column “ADvsRES” denotes genes that were also differentially expressed between AD and resilient subjects (related with **Table S1**).

Table S6. Dysregulated pathways in AD compared to cognitive resilience in bulk DLPFC.

Differential pathway activity analysis was performed between AD and resilient subjects based on ROSMAP bulk RNAseq data. Significant pathway dysregulation was determined using

bioRxiv preprint doi: https://doi.org/10.1101/2025.01.13.632801; this version posted January 15, 2025. The copyright holder for this preprint (which was not certified by peer review) is the author/funder, who has granted bioRxiv a license to display the preprint in perpetuity. It is made available under aCC-BY 4.0 International license.

Storey-adjusted p-value (q-value) < 0.1. P-values were determined using a linear regression model that accounts for confounding covariates such as sequencing batch, RNA integrity number, and postmortem interval.

Table S7. Unsupervised clusters of dysregulated pathways in AD compared to cognitive resilience from bulk DLPFC.

Cluster membership for each of the 99 dysregulated pathways in ADvsRES (related to **Table S6**) following unsupervised clustering. Modules of dysregulated pathways in ADvsRES were determined by mapping to the pathway co-expression network followed by the Label Propagation clustering algorithm. The column “cluster” represents the unsupervised clusters assigned by the described analysis. The analysis was performed using the PanomiR package with default parameters.

Table S8. Dysregulated pathways in AD compared to cognitively healthy controls in bulk DLPFC.

Differential pathway activity analysis was performed between AD and control subjects based on ROSMAP bulk RNAseq data. Significant pathway dysregulation was determined using Storey-adjusted p-value (q-value) < 0.1. p-values were determined using a linear regression model accounting for confounding covariates such as sequencing batch, RNA integrity number, and postmortem interval.

Table S9. Results for the pathway activity analysis in resilient individuals compared to subjects classified as presymptomatic in bulk DLPFC.

Differential pathway activity analysis was performed between RES and presymptomatic subjects (RESvs PRE) based on ROSMAP bulk RNAseq data. Significant pathway dysregulation was determined using Storey-adjusted p-value (q-value) < 0.1. P-values were determined using a

bioRxiv preprint doi: https://doi.org/10.1101/2025.01.13.632801; this version posted January 15, 2025. The copyright holder for this preprint (which was not certified by peer review) is the author/funder, who has granted bioRxiv a license to display the preprint in perpetuity. It is made available under aCC-BY 4.0 International license.

linear regression model that accounts for confounding covariates such as sequencing batch, RNA integrity number, and postmortem interval.

Table S10. Results for the pathway activity analysis in AD compared to presymptomatic subjects in bulk DLPFC tissue.
Differential pathway activity analysis was performed between AD and presymptomatic subjects (ADvs PRE) based on ROSMAP bulk RNAseq data. Significant pathway dysregulation was determined using Storey-adjusted p-value (q-value) < 0.1. P-values were determined using a linear regression model that accounts for confounding covariates such as sequencing batch, RNA integrity number, and postmortem interval.

Table S11. Number of subjects and number of cells per group defined in this study for snRNAseq data.

Table S12. Distributions for differentially expressed genes per comparison, identified from snRNAseq data for each major cell type in each brain region investigated.
Differential gene expression analyses were performed per major cell type for each brain region by implementing a statistical model by group using the MAST statistical framework in *Seurat*, after removing non-variable genes. P-values were adjusted using the Bonferroni correction, as recommended for the R package *Seurat*. Significant differential expression was determined using adjusted p-value (adj-P) < 0.1 and log2FC > 0.2 or log2FC < -0.2 (|FC| > 1.1) criteria. The number of down-regulated differentially expressed genes (DEGs) in the first group compared to the second group are highlighted in red, and the number of up-regulated DEGs in the first group compared to the second group are highlighted in blue.

Table S13. Selected differentially expressed genes for major cell types.

bioRxiv preprint doi: https://doi.org/10.1101/2025.01.13.632801; this version posted January 15, 2025. The copyright holder for this preprint (which was not certified by peer review) is the author/funder, who has granted bioRxiv a license to display the preprint in perpetuity. It is made available under aCC-BY 4.0 International license.

Differential expression summary results for each brain region across the three comparisons (ADvsRES, ADvsCTRL, and RESvsCTRL) for genes discussed in the text. Differential gene expression analyses were performed per major cell type for each brain region by implementing a statistical model by group using the MAST statistical framework in *Seurat*, after removing non-variable genes. P-values were adjusted using the Bonferroni correction (adj-P), as recommended for the R package *Seurat*. Significant differential expression was determined using adjusted adj-p < 0.1 and log2FC > 0.2 or log2FC < -0.2 (|FC| > 1.1) criteria. NS: not significant. Direction "NONE" refers to log2FC below our threshold.

Table S14. Cell annotations for cell subtypes for each brain region.

Cluster annotations were generated using the web tool MapMyCells using the Hierarchical algorithm.

Table S15. Results for cell proportion analysis per major cell types for each brain region investigated.

Changes in cell composition between groups for each cell subpopulation (subclusters) were detected using a Dirichlet multinomial regression model, while accounting for the proportions of all of the other cell subsets within each major cell type. P-values were adjusted for multiple comparisons using the Benjamini-Hochberg (FDR) correction (adj-P). Green: Adj-P < 0.05, yellow: adj-P < 0.01, red: adj-p < 0.001.

Table S16. Phenotypic and clinical characteristics of the BIDMC cohort.

Human brain tissue used for immunostaining experiments was collected at Beth Israel Deaconess Medical Center (BIDMC) upon autopsy. Samples were tested for multiple pathologies, including TDP-43. Donors presenting comorbidities, including diabetes, were excluded.

bioRxiv preprint doi: https://doi.org/10.1101/2025.01.13.632801; this version posted January 15, 2025. The copyright holder for this preprint (which was not certified by peer review) is the author/funder, who has granted bioRxiv a license to display the preprint in perpetuity. It is made available under aCC-BY 4.0 International license.

Table S17. Ligand-receptor results from CellChat for the SST signaling pathway in the EC.

Source cell subtypes included EC:Inh3 and EC:Inh9 in control and resilient subjects, but in AD only EC:Inh3 and EC:Inh11 were identified as sources for the SST pathway in AD. The receptor SSTR1 was only observed in resilience.

Table S18. Selected differentially expressed genes for cell subtypes.

Differential expression summary results for each brain region across the three comparisons (ADvsRES, ADvsCTRL, and RESvsCTRL) for genes discussed in the text. Differential gene expression analyses were performed per cell subtype for each brain region by implementing a statistical model by group using the MAST statistical framework in *Seurat*, after removing non-variable genes. P-values were adjusted using the Bonferroni correction (adj-P), as recommended for the R package *Seurat*. Significant differential expression was determined using adjusted adj-p < 0.1 and log2FC > 0.2 or log2FC < -0.2 (|FC| > 1.1) criteria. NS: not significant. Direction “NONE” refers to log2FC below our threshold.

Table S19. Interaction partners of RBFOX1 identified as rare variant-associated genes and marker genes for DLPFC:Inh1 neurons

Table S20. Functional enrichment of RBFOX1 partners, also identified as rare variant associated genes and marker genes for DLPFC:Inh1 neurons.
